# The fate of bromine after temperature-induced dehydrogenation of on-surface synthesized bisheptahelicene†
†Electronic supplementary information (ESI) available. See DOI: 10.1039/c8sc04720k


**DOI:** 10.1039/c8sc04720k

**Published:** 2019-01-15

**Authors:** Anaïs Mairena, Milos Baljozovic, Maciej Kawecki, Konstantin Grenader, Martin Wienke, Kévin Martin, Laetitia Bernard, Narcis Avarvari, Andreas Terfort, Karl-Heinz Ernst, Christian Wäckerlin

**Affiliations:** a Empa, Swiss Federal Laboratories for Materials Science and Technology , 8600 Dübendorf , Switzerland . Email: christian.waeckerlin@empa.ch ; Email: karl-heinz.ernst@empa.ch; b Department of Chemistry , Institute of Inorganic and Analytical Chemistry , Goethe-University , 60438 Frankfurt , Germany; c Department of Chemistry , University of Hamburg , 20146 Hamburg , Germany; d Laboratoire Moltech-Anjou , CNRS-Université d'Angers , 49045 Angers , France; e Department of Chemistry , University of Zurich , 8057 Zurich , Switzerland

## Abstract

The on-surface synthesis of bisheptahelicene by Ullmann coupling of 9-bromoheptahelicene on Au(111) and its temperature-induced dehydrogenation is studied using temperature-programmed reaction spectroscopy and time-of-flight secondary ion mass spectrometry. Specific dehydrogenation products of bisheptahelicene after loss of 6, 8 and 10 hydrogen atoms are identified, corresponding to molecules having undergone Diels–Alder transformations and intramolecular C–C coupling reactions. By combining with atomic hydrogen produced by dehydrogenation, the Ullmann coupling side-product bromine desorbs as HBr. H_2_ desorption emerges only after all Br has desorbed. Such characteristic behavior is explained by a kinetic model which explicitly considers the coverage of transient atomic H on the surface. Heating experiments performed with saturated layers of different Br-containing molecules reveal that the onset of HBr desorption depends strictly on the dehydrogenation step and therefore on the structure of the molecules.

## Abstract 2

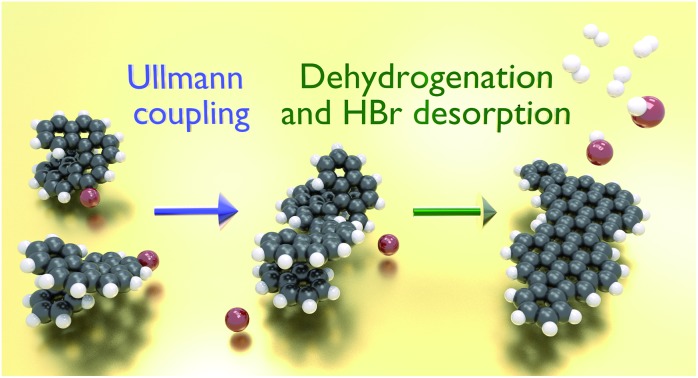
The dehydrogenation of bisheptahelicene leads to specific products and induces desorption of the side-product bromine as hydrogen bromide.

## Introduction

A very broad range of oligomers, 1D and 2D polymers can be obtained reliably and predictably by Ullmann coupling on surfaces.^1^–^6^ The halogen atom (typically Br or I) remains adsorbed on the surface as side-product after the reaction. In a second step, the on-surface synthesized hydrocarbon products can be modified by intra- or intermolecular dehydrogenation.^6^–^12^ Depending on the structural changes induced by this second dehydrogenation step it may be very difficult to identify the reaction products with common surface analytical methods. *In situ* time-of-flight secondary ion mass spectrometry (ToF-SIMS) is a very valuable tool to precisely identify reaction products, but has been rarely applied to unravel on-surface chemistry. In combination with temperature-programmed reaction spectroscopy (TPRS), volatile desorption products as well as the remaining adsorbates can be analysed using mass spectrometry, thus providing deeper insight into the reaction landscape.

Because of its low barrier for recombinative desorption on Cu, Ag and Au,^13^ it is expected that the adsorbed atomic hydrogen desorbs immediately after dehydrogenation and is therefore an irrelevant side-product. However, recent work showed that atomic hydrogen produced by on-surface chemistry, such as dehydrogenation or metalation-induced dehydrogenation, may react with other species instead of desorbing.^14^–^20^ Recently, desorption of HBr during cyclodehydrogenation of polymerized 10,10′-dibromo-9,9′-bianthryl (DBBA) on Au(111)^14^ and of polymerized 1,6-dibromopyrene on Ag(110)^19^ has been reported. It has been shown that Br and I, initially bound to the otherwise identical precursor, leave the surface at the same temperature.^20^ The kinetics of this important reaction in Ullmann-chemistry based on-surface synthesis is not entirely understood. The true desorption temperature of pure Br/Au(111) is unknown and the reason for the large variations in the reported temperatures (500 to 633 K) for Br desorption from Au(111) in presence of different hydrocarbons^20^–^25^ remains unclear.

**Fig. 1:**
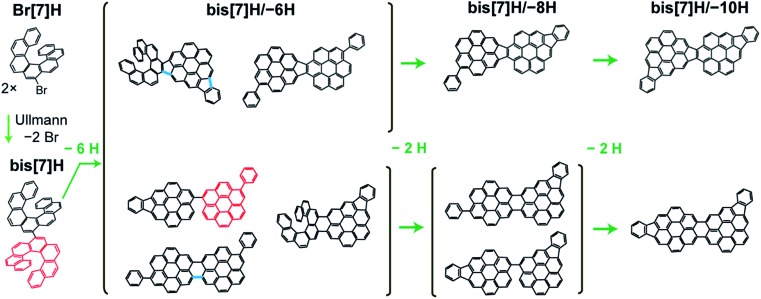
Potential reaction pathways for dehydrogenation of bis[7]H after Ullmann coupling of Br[7]H. A subset of stereomers is shown to illustrate potential reaction products in the dehydrogenation process due to steric overcrowding. A Diels–Alder reaction causes the transformation of a [7]H into a phenylcoronene subunit (exemplified in red, loss of 2 H per [7]H unit). Cyclodehydrogenation between the helicenes and at the phenylcoronene creates new C–C bonds, resulting in indenocoronene subunits or new 5- or 6-membered rings (loss of 2 H atoms per new C–C bond). Some of these new bonds are illustrated in blue. Different combinations of the aforementioned processes lead to the loss of 2, 4, 6, 8 and 10 H atoms. Experimentally, isomers having lost 6, 8 and 10 H atoms are identified.

Here, the Ullmann coupling of 9-bromoheptahelicene (Br[7]H) to bisheptahelicene (bis[7]H) on Au(111) in ultrahigh vacuum and the following temperature-induced cyclodehydrogenation of bis[7]H (Fig. 1) are studied using TPRS and ToF-SIMS. For comparison, the dehydrogenation chemistry is also studied for solution-synthesized bis[7]H deposited onto the gold surface. A simple kinetic model, which explicitly considers the coverage of transiently existing atomic H, is introduced to explain HBr and H_2_ desorption spectra. The different desorption temperatures of Br as HBr of different molecules are compared and are shown to be significantly lower than the desorption temperature of Br from Au(111) in the absence of molecules.

## Experimental

The samples are prepared in ultra-high vacuum by Ar^+^ sputtering and annealing of Au(111) followed by the sublimation of respective molecules and analysed *in situ* by TPRS and ToF-SIMS. The sample temperature is measured by a thermocouple directly attached to the crystal. The heating is performed resistively (ToF-SIMS) and by electron-beam bombardment (TPRS) to the backside of the sample holder.

### TPRS measurements

TPRS is measured using a quadrupole mass spectrometer (Balzers QME 200) after preparing the samples *in situ.* The base pressure was better than 10^–9^ mbar. The MS signal recorded before starting the temperature ramp, which corresponds to the partial base pressure, is subtracted.

### ToF-SIMS measurements

ToF-SIMS (IONTOF ToF-SIMS 5) is performed with a beam of 25 keV Bi_3_^+^ used as primary ions. The spectra were acquired by randomly rastering the beam of Bi_3_^+^ ion pulses over the area of 500 × 500 μm^2^. An extraction voltage of 3 kV was used. The total dose density is kept below 10^12^ ions per cm^2^, *i.e.* in the static limit. A new spot on the sample is analysed for each annealing step. 100 scans in each polarization (positive and negative) were collected at the same spot. The spectra obtained within those 100 scans were identical to the first and last 10 scans (Fig. S3†), evidencing that the detected signals are not affected by the Bi_3_^+^ bombardment. The pressure in the chamber during preparation and measurements was below 10^–8^ mbar.

### Deposition of Br

**Fig. 2:**
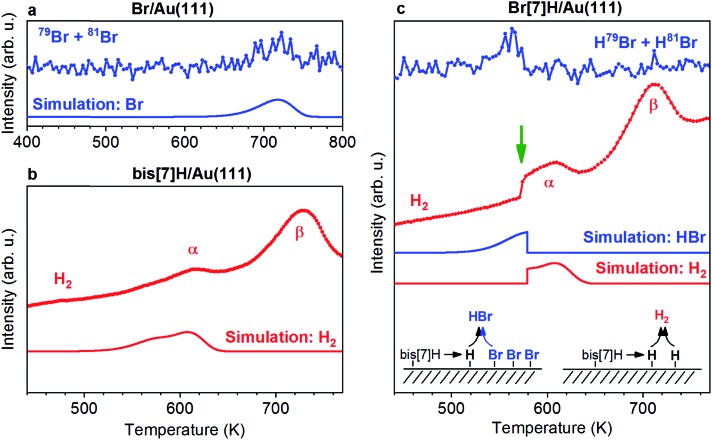
Experimental and simulated TPR spectra of adsorbates on Au(111). (a) Br TPR spectra of Br/Au(111). In the absence of other species, Br desorbs at 720 K. (b) H_2_ TPR spectra of 0.87 ML bis[7]H/Au(111). Two peaks are observed; α corresponds to cyclodehydrogenation/Diels–Alder cycloaddition including dehydrogenation and β to further non-specific dehydrogenation. Only the relevant peak α is modelled. (c) Br and H_2_ TPR spectra of 1 ML Br[7]H/Au(111). Simulations considering competing desorption of hydrogen as H_2_ and HBr reproduce the step like feature (green arrow). The heating rate is 3 K s^–1^.

Br is deposited by annealing AuBr_3_ in a quartz crucible to 373 K while the Au(111) substrate is kept at 473 K. No Au is detected with XPS after deposition on Cu(111) kept at room temperature. Thus we conclude that AuBr_3_ decomposes and releases Br_2_ or Br. Deposition on Au(111) kept at 473 K for 3 min results in a very similar Br coverage (detected by XPS, Fig. S1†) as in case of 1 ML of Br[7]H. Deposition on the sample kept at room temperature leads to much higher Br coverages. As shown in ref. 29 and 30, the high coverage phases have lower desorption temperatures than the low coverage phase shown in Fig. 2a.

### Deposition of molecules

The molecules are deposited by sublimation onto the sample kept at room temperature. 1 ML refers to a saturated layer. For Br[7]H (9-bromoheptahelicene) and Br[4]H (2-bromotetrahelicene), 1 ML is obtained by desorption of the multilayer by annealing to 373 K and 413 K respectively. The coverage of bis[7]H (bisheptahelicene) and diBr[4]H (2,3-dibromo[4]helicene) is defined by comparing the C 1s XP signal with 1 ML of Br[7]H and Br[4]H, respectively. In the case of BrP (1-bromopyrene), the adsorption of molecules beyond the first layer is self-limiting at room temperature. The corresponding C 1s XP spectra are shown in Fig. S2.†

### Synthesis

Br[7]H, bis[7]H, Br[4]H and diBr[4]H were synthesized according to the literature.^26^–^28^ DBBA and BrP were purchased from Sigma-Aldrich.

## Results and discussion

### H_2_ and HBr desorption

Fig. 2 shows TPR spectra of saturated monolayers of bis[7]H and Br[7]H on Au(111) as well as of a submonolayer of Br equivalent to the amount of Br present in a saturated layer of Br[7]H. In the absence of molecules, Br desorbs at 720 K (Fig. 2a). No HBr signal is detected. In view of the desorption of Cl, Br and I from Ag and Cu surfaces as metalhalides,^29^–^31^ and the proposed likely desorption I on Au(111) as AuI,^20^ desorption as AuBr should be considered. If this is the case, the detected Br signal is due to fragmentation in the mass spectrometer. Unfortunately, the high mass of AuBr does not allow for its detection by our instrumentation. In the simple picture of first order kinetics and assuming a pre-exponential factor of 10^13^ Hz, an activation energy for desorption of 192 kJ mol^–1^ is derived (Fig. 2a, see also ESI†). This value is significantly lower than the adsorption energies of Br (270 kJ mol^–1^) and Br_2_ (296 kJ mol^–1^) on Au(111) calculated by DFT,^32^ indicating that Br desorbs indeed as AuBr.

In the H_2_ TPR spectrum of bis[7]H (Fig. 2b), two peak maxima α and β are observed. The continuously rising H_2_ background is due to a general warming up of the sample holder. Signal α is identified as dehydrogenation due to steric overcrowding, in accordance with the literature^6^,^14^,^18^ and with the ToF-SIMS results reported below. Peak β corresponds to non-specific dehydrogenation.^18^,^33^

**Fig. 3:**
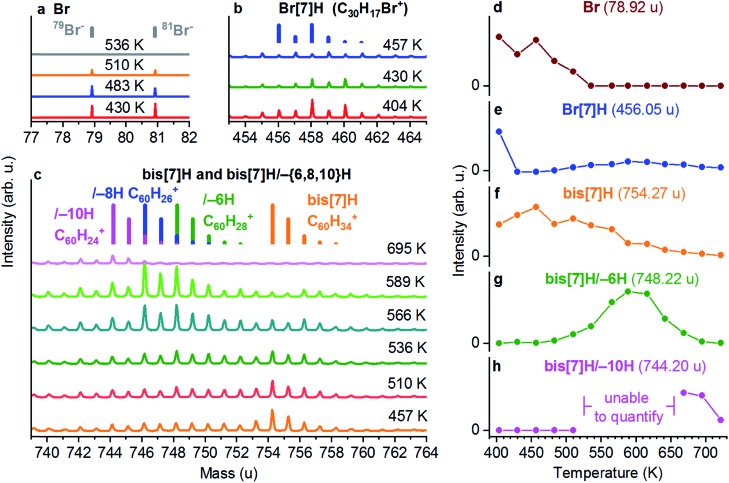
ToF-SIMS analysis of a saturated layer of Br[7]H on Au(111). The saturated layer was obtained by desorption of excess molecules from a multilayer by annealing to 404 K. (a) Br^–^, (b) Br[7]H (C_30_H_17_Br^+^) and (c) C_60_H_{34,28,26,24}_^+^ corresponding to bis[7]H/–{6,8,10}H signals. The mass distributions of C_60_H_{34,28,26,24}_^+^ are shown as coloured bars. Integrated intensities of the main peak of (d) Br, (e) Br[7]H, (f) bis[7]H, (g) bis[7]H/–6H and (h) bis[7]H/–10H. Annealing to 404 K already induces partial Ullmann coupling to bis[7]H (e). Ullmann coupling is complete after annealing to 457 K (e). The onset of dehydrogenation occurs after annealing to 510 K (g) and coincides with the vanishing Br signal (d). The intensity of bis[7]H/–6H (and bis[7]H/–8H which is not quantified) reaches its maximum at 589 K. After annealing to 695 K, only bis[7]H/–10H is detected.

The H_2_ TPR spectrum obtained for 1 ML Br[7]H (Fig. 2c) is very similar to the one for bis[7]H, with the exception that the first part of peak α is missing and that the H_2_ desorption rate rises suddenly at 574 K (green arrow). The HBr desorption rate peaks at 563 K and drops to zero at 574 K. This desorption temperature of Br in the presence of [7]helicenes is 160 K lower than for Br in absence of molecules. ToF-SIMS shows complete coupling of Br[7]H to bis[7]H after annealing to 457 K (Fig. 3). Hence, Br[7]H has already undergone Ullmann coupling in the temperature range of peak α. The conspicuous step in the H_2_-TPRS trace and formation of HBr before onset of H_2_ evolution strongly suggests that HBr desorption is induced by the dehydrogenation of bis[7]H. Consequently, depletion of Br from the Au surface goes hand in hand with dehydrogenation of the hydrocarbons.

### Dehydrogenation products identified by SIMS

The chemical transformation of Br[7]H on the basis of the ToF-SIMS data is shown in Fig. 3. It displays ToF-SIMS spectra taken from a multilayer of Br[7]H/Au(111) annealed sequentially to different temperatures. The full sequence is shown in Fig. S4.† Br^–^ ions (Fig. 3a), C_30_H_17_Br^+^ ions (Fig. 3b) and C_60_H_{34,28,26,24}_^+^ ions (Fig. 3c) are indicative for Br, Br[7]H, bis[7]H and its dehydrogenation products, respectively. Annealing to 404 K leads to desorption of molecules from the second layer (Fig. S4†). In particular for the assignment of the bis[7]H-related signals the characteristic mass distribution, due to ^13^C, allows for an unambiguous identification (indicated as bars in Fig. 3a–c). The presence of a bis[7]H signal after annealing to 404 K indicates that some of the Br[7]H molecules have already undergone Ullmann coupling (Fig. 3c). Annealing to 457 K leads to the disappearance of the Br[7]H-based signals, evidencing complete debromination and Ullmann coupling (Fig. 3b).

This observation is consistent with coupling temperatures (380 to 473 K) reported for other brominated molecules on Au(111).^4^,^34^–^36^ The remaining signals correspond to the SIMS-induced fragmentation pattern of bis[7]H, spreading over a large mass range below its main peak. For example, after complete coupling of Br[7]H at 457 K, the intensity at the main peak of Br[7]H due to fragmentation is 9.5% of the bis[7]H signal (Fig. S4c†). Therefore, in Fig. 3e, which shows the integrated intensity of the main peak of Br[7]H as a function of temperature, 9.5% of the intensity of the bis[7]H signal is subtracted. Partial Br desorption occurs after annealing to 510 K and is complete after annealing to 536 K. In addition, starting from 510 K, the signals in the bis[7]H mass range shift towards lower masses (Fig. 3c). In particular C_60_H_28_^+^ (bis[7]H/–6H), C_60_H_26_^+^ (bis[7]H/–8H) and at higher temperatures C_60_H_24_^+^ (bis[7]H/–10H) signals are detected. The onset of dehydrogenation coincides with the reduction of the Br intensity (Fig. S4a and c†).

In order to analyse changes in the intensity of the bis[7]H/–6H peak, the fragmentation pattern of bis[7]H is taken into account (Fig. 3g) in the same way as for Br[7]H. The intensity of bis[7]H/–8H is not analysed quantitatively, because fragmentation of both bis[7]H/–6H and bis[7]H would need to be taken into account. Qualitatively it can be said that the intensity of bis[7]H/–8H closely follows the one of bis[7]H/–6H. For the same reason, bis[7]H/–10H is analysed only in the temperature ranges where the intensities of signals related to bis[7]H/–6H and bis[7]H/–8H are negligible (Fig. 3h). The species of bis[7]H/–10H appears at higher temperatures than bis[7]H/–6H and bis[7]H/–8H and remains the sole specific bis[7]H product at 695 K (Fig. 3c). Annealing to 722 K leads to disappearance of all bis[7]H-related signals (Fig. S4c†). The intensity of the C^–^ signal does not change significantly during these steps (data not shown). Therefore, the loss of specific masses is due to non-specific C–C coupling and fragmentation leading to many different products and not due to desorption of significant amounts of carbon.

ToF-SIMS data obtained for a submonolayer coverage (≈0.25 ML) of solution-synthesized bis[7]H (Fig. S5†), compared with ≈0.25 ML of Br[7]H (Fig. S6†) show no significant differences in the dehydrogenation reactions. This confirms that the presence of Br does not influence the dehydrogenation process. There are slight differences with respect to the saturated layer of Br[7]H: the temperatures for Ullmann coupling, Br desorption (after debromination of Br[7]H) and dehydrogenation reactions are slightly lower. Moreover, the Br[7]H spectrum at high coverage exhibits peaks corresponding to C_30_H_19_Br^+^, *i.e.* Br[7]H with two additional H atoms. Since these higher masses are also observed in the non-annealed Br[7]H multilayer (Fig. S4†), but not in the dilute submonolayer (Fig. S6†), it is tentatively ascribed to SIMS related hydrogenation during desorption of the secondary ions.^37^

The temperature-induced transformation of dibenzoheptahelicene (DBH, dinaphtho[2,1;1′,2′-*f*,*j*]picene) on Ag(111) has been studied in great detail previously by Stetsovych *et al.*^12^ Planarization of DBH to a dibenzocoronene occurs *via* Diels–Alder cycloaddition and a phenyl group shift at 520 K, followed by dehydrogenation and C–C bond formation between phenyl group and the dibenzocoronene unit. Each of these two steps releases 2 H atoms. Starting from bis[7]H or Br[7]H, respectively, the discussed reactions predict bis[7]H/–{2,4,6,8,10}H as possible products (Fig. 1). The Diels–Alder cycloaddition and phenyl group shift of the 7[H] subunit and the C–C bonds formed by intramolecular dehydrogenation are exemplified in red and blue, respectively. After losing 10 H, which is equivalent to formation of 5 new C–C bonds, the molecule is completely planar with no possibility for further intramolecular cyclodehydrogenation. The observation of masses corresponding to bis[7]H/–{6,8,10}H confirms such scenario. Interestingly, bis[7]H/–{2,4}H was not detected; the first dehydrogenation product is bis[7]H/–6H. This may be explained by planarization *via* Diels–Alder at one helicene subunit, which allows for stronger interaction with the substrate, increasing the driving force for further dehydrogenation. In particular the planar isomers (Fig. 1) are expected to be the most probable bis[7]H/–6H isomers. A side-step in which benzyne and H_2_ extrusion occurs (–78 u)^12^ is not observed here on Au(111).

### Mechanism of HBr and H_2_ desorption

HBr and H_2_ desorption are modelled based on a generalized version of the Polanyi–Wigner equation.^38^,^39^ Because the Ullmann coupling occurs well before the dehydrogenation and HBr desorption steps, the simulation starts with bis[7]H and Br rather than Br[7]H. The rate equations (see ESI†) explicitly model the coverage of transient atomic H (*θ*_H_). The temperature position of the sudden rise in the H_2_ TPR spectra with respect to the H_2_ TPR maxima is determined by the number of bromine atoms with respect to the number of released hydrogen atoms (Fig. S5†). H_2_ and HBr TPR spectra simulated with 2 Br atoms per bis[7]H and 6H atoms produced by dehydrogenation reproduce the temperature of the step quite well (Fig. 3c). The loss of 6 to 10 H per molecule (Fig. S6†) is consistent with the dehydrogenation products identified by ToF-SIMS.

**Fig. 4:**
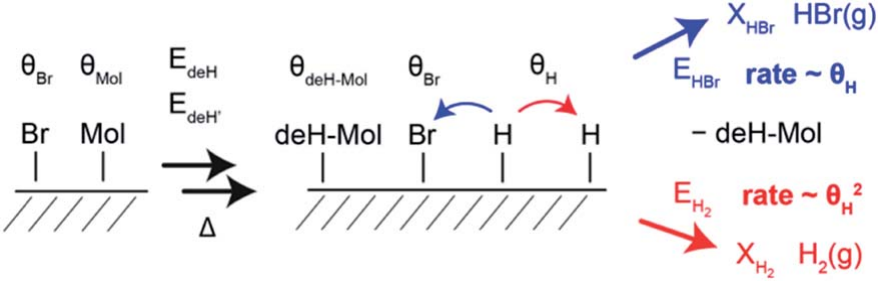
Sketch of the dehydrogenation reaction inducing H_2_ and HBr desorption. In presence of Br, the atomic H produced by dehydrogenation can either desorb as H_2_ or HBr. Because the rates of H_2_ and Br desorption are much higher than the rate of atomic hydrogen production, the coverage by atomic H (*θ*_H_) is very small at any time. Since HBr desorption is first order with respect to atomic hydrogen and H_2_ desorption is second order, H_2_ desorption is strongly disfavoured until Br has been completely desorbed.

Based on the simulations, the mechanism of HBr and H_2_ evolution can be described as follows: because the rate limiting step is the dehydrogenation and the two competing reaction pathways (H_2_ and HBr desorption) have similarly low activation energies,^14^ any produced atomic H can immediately react with surface adsorbed Br and desorbs as HBr (Fig. 4). This reaction is first order with respect to the coverages of atomic H and Br and its rate is proportional to the product of the coverages *θ*_H_ × *θ*_Br_. The H_2_ desorption pathway requires collision of two surface adsorbed hydrogen atoms and is therefore a second order process. The rate of H_2_ desorption is therefore proportional to *θ*_H_^2^. Because the first process keeps the coverage of transient atomic H extremely low over a wide range of Br coverage, the H_2_ desorption is enabled suddenly once the Br coverage reaches zero, leading to the conspicuous step. At temperatures at which peak α is observed, the HBr desorption rate is about seven orders of magnitude higher than the rate of dehydrogenation. Therefore, the model is not sensitive for changes of the attempt frequencies and the exact values of the activation energies for H_2_ and HBr desorption are not critical. With the expected H/Br ratio of 4, the model used here also reproduces the step of the H_2_ TPRS of ref. 14 and its temperature position within the H_2_ desorption peak (Fig. S8†).

### General trend for HBr desorption in presence of different hydrocarbons

**Fig. 5:**
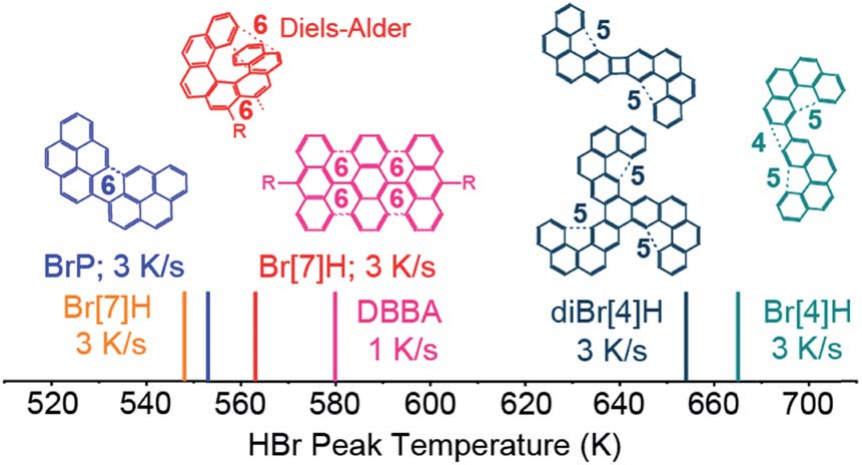
TPRS HBr desorption maxima of saturated layers of BrP, Br[7]H, DBBA,^14^ diBr[4]H and Br[4]H on Au(111). The desorption temperature varies by more than 100 K. The structures of the Ullmann coupling products which are relevant for the dehydrogenation reactions are shown. As indicated by dashed lines, bispyrene, polyanthracene^6^ and bis[7]H can intramolecularly (cyclo)dehydrogenate to unstrained C_6_-rings, while bis[4] and tris[4]helicenes cannot form C_6_ rings, but strained C_4_ and C_5_-motifs or undergo possibly other intermolecular C–C coupling and dehydrogenation.

In order to understand why the reported temperatures for Br desorption vary by more than 100 K,^20^–^25^ HBr desorption from saturated monolayers of 1-bromopyrene (BrP), 2,3-dibromo[4]helicene (diBr[4]H) and 2-bromo[4]helicene (Br[4]H) on Au(111) is studied (Fig. 5). The respective TPR spectra are shown in Fig. S9.† The HBr peak temperature for DBBA on Au(111)^14^ is also included in Fig. 5. For the different molecules, the HBr desorption temperatures span from 548 K to 665 K. For molecules which can form C_6_-rings (BrP, DBBA and Br[7]H) after on-surface Ullmann coupling,^6^,^26^,^36^ HBr desorbs in the range of 548 to 580 K.

For bis- and tris[4]helicenes obtained by Ullmann coupling of Br[4]H and diBr[4]H,^26^,^36^ HBr desorption occurs at 653 K and 665 K. Here, the possible dehydrogenation products involve the formation of less-favoured 4- or 5-membered rings. At such high temperatures, dehydrogenation by intermolecular C–C coupling cannot be excluded. This tentative model qualitatively explains the higher dehydrogenation temperatures of tetrahelicene dimers and trimers with respect to bis-pyrene, bis-heptahelicene and polyanthracene.

## Conclusions

In conclusion, starting from Br[7]H, the step-wise Ullmann coupling, dehydrogenation and HBr desorption are studied using mass spectrometry. ToF-SIMS reveals the dehydrogenation of bisheptahelicenes losing 6, 8 and 10 H atoms. Such products correspond to a reaction mechanism proposed previously for dibenzoheptahelicene.^12^ Comparison to experiments with *ex situ* synthesized bis[7]H as starting material, rather than Br[7]H, reveals that Br does not affect the dehydrogenation process. TPRS simulations assuming low activation energies for HBr and H_2_ desorption explain the peculiar characteristics of the HBr and H_2_ TPR signals. HBr desorption as a first order reaction with respect to the atomic H concentration is favoured over the H_2_ desorption as a second order reaction. Because the dehydrogenation reaction is the rate limiting step, the concentration of atomic H is extremely low as long as Br is present. Therefore, H_2_ desorption is enabled exactly when all Br is desorbed. Different HBr desorption temperatures are explained by different dehydrogenation mechanisms of the Ullmann-coupling products of different brominated polycyclic aromatic hydrocarbons. A strong correlation between the structure of the dehydrogenation product and the HBr desorption temperature is found. For molecules for which the formation of C_6_ rings is possible, HBr desorption occurs at around 560 K. In the case of molecules allowing only C_4_ and C_5_ rings formation or intermolecular dehydrogenation, HBr desorption at an about 100 K higher temperature is observed. Consequently, a connection between stereochemistry of hydrocarbons and HBr desorption temperature is established.

## Conflicts of interest

There are no conflicts to declare.

## Supplementary Material

Supplementary information
