# Supplementary material for: The fate of bromine after temperature-induced dehydrogenation of on-surface synthesized bisheptahelicene
Source: Chem Sci. 2019 Jan 15;10(10):2998–3004. doi: 10.1039/c8sc04720k (PMC6430192; doi:10.1039/c8sc04720k)
Supplement: Supplementary file 1 [file SC-010-C8SC04720K-s001.pdf]

Electronic Supplementary Material (ESI) for Chemical Science.
This journal is © The Royal Society of Chemistry 2019

Electronic Supporting Information:

The fate of bromine after temperature-induced dehydrogenation of on-surface synthesized bisheptahelicene

Anaïs Mairena[a], Milos Baljozovic[a], Maciej Kawecki[a], Konstantin Grenader[b], Martin Wienke[c], Kévin Martin[d], Laetitia Bernard[a], Narcis Avarvari[d], Andreas Terfort[b], Karl-Heinz Ernst[a,e,] and Christian Wäckerlin[a,*]*

[a] Empa, Swiss Federal Laboratories for Materials Science and Technology, 8600 Dübendorf, Switzerland, E-mail: christian.waeckerlin@empa.ch, karl-heinz.ernst@empa.ch

[b] Department of Chemistry, Institute of Inorganic and Analytical Chemistry, Goethe-University, 60438 Frankfurt, Germany

[c] Department of Chemistry, University of Hamburg, 20146 Hamburg, Germany

[d] Laboratoire Moltech-Anjou, CNRS-Université d'Angers, 49045 Angers, France

[e] Department of Chemistry, University of Zurich, 8057 Zurich, Switzerland

Contents

TPRS Models 3
Supplementary Figures 4
References 12

TPRS Models

Equation S1 describes a simple first order process, and is used for example to model the desorption of Br. All rate equations are evaluated on a linear temperature ramp $T(t) = \xi t$. ξ corresponds to the heat rate. For simplicity, the attempt frequency of ν = 10^{13} Hz is used throughout the modelling. r_{Br} corresponds to the desorption rate of Br and is compared with the experimental data (Figure 2a).

$$-\mathrm{r_{Br}}(\mathrm{t}) = \frac{d\theta_{Br}}{dt} = -\nu\,\theta_A\,e^{-\frac{E_{Br}}{RT}}\ (\mathrm{S1})$$

Equations S2 – S6 describe the evolution of molecular coverage (S2), dehydrogenation of molecules (S3), coverage of atomic H (S4), the coverage and rate of desorbing HBr (S5) and the rate of H_2 (S6). θ_{mol} and θ_{moldeH} describe the coverages of molecules (here bis[7]H) and dehydrogenated molecules. Θ_{Br} and Θ_{Hat} describes the coverage of adsorbed Br and atomic H. r_{HBr} and r_{H2} correspond to the rates of desorbing HBr and H_2.

In order to reproduce the broad H_2 TPRS signal of bis[7]H (Figure 2b), the dehydrogenation has been modelled in two steps (E_{deh} = 150 kJ mol^{-1}, $E_{deh'}$ = 161.5 kJ mol^{-1}). 2 and 4 H atoms are released in the two respective steps. The activation energies for H_2 and HBr desorption (E_{H2} = 66.2 kJ mol^{-1} and E_{HBr} = 71.0 kJ mol^{-1}) were taken from ref. 1.

$$\frac{d\theta_{mol}}{dt} = -\nu\,\theta_{mol}\,e^{-\frac{E_{deH}}{RT}}\ (\mathrm{S2})$$

$$\frac{d\theta_{moldeH}}{dt} = +\nu\,\theta_{mol}\,e^{-\frac{E_{deH}}{RT}} - \nu\,\theta_{moldeH}\,e^{-\frac{E_{deH'}}{RT}}\ (\mathrm{S3})$$

$$\frac{d\theta_{Hat}}{dt} = +2\,\nu\,\theta_{mol}\,e^{-\frac{E_{deH}}{RT}} + 4\,\nu\,\theta_{moldeH}\,e^{-\frac{E_{deH'}}{RT}} - \nu\,\theta_{Br}\,\theta_{Hat}\,e^{-\frac{E_{HBr}}{RT}} - 2\nu\,{\theta_{Hat}}^2 e^{-\frac{E_{H_2}}{RT}}\ (\mathrm{S4})$$

$$-\mathrm{r_{HBr}}(\mathrm{t}) = \frac{d\theta_{Br}}{dt} = -\nu\,\theta_{Br}\,\theta_{Hat}\,e^{-\frac{E_{Br}}{RT}}(\mathrm{S5})$$

$$r_{H_2}(t) = \nu\,{\theta_{Hat}}^2 e^{-\frac{E_{H_2}}{RT}}(\mathrm{S6})$$

Supplementary Figures

The XP spectra shown in Fig. S1 and S2 were obtained with non-monochromatic Al K_{α} X-rays in normal incidence. The spectra are normalized with respect to the intensity of the $Au4f_{7/2}$ peak.

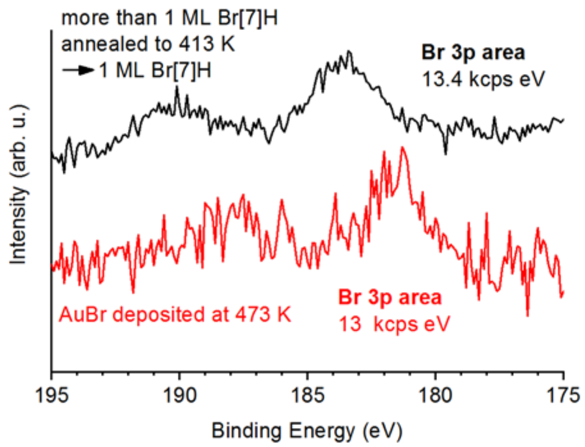

Figure S1: Br 3p XP spectra of 1 ML Br[7]H prepared by desorption of second layer molecules compared with Br deposited by decomposition of AuBr. The integrated Br 3p intensities are very similar. The difference in binding energy is due the presence of C-Br bonds in Br[7]H and Br-Au bonds in Br deposited by decomposition of AuBr.[2] The spectra are offset in intensity for clarity.

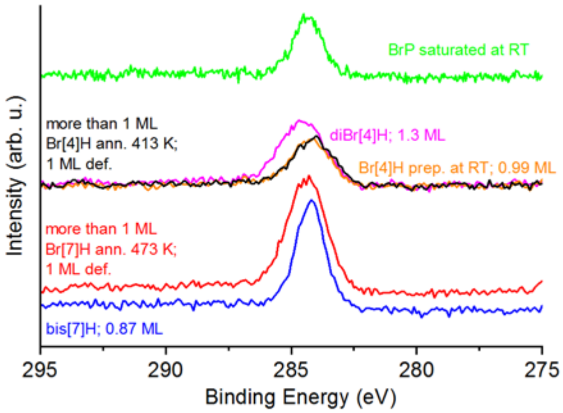

Figure S2: C 1s XP spectra of Br[7]H, bis[7]H, diBr[4]H, Br[4]H and BrP. The spectra are offset in intensity for clarity. As described in the methods section, saturated 1 ML coverages of Br[7]H

and Br[4]H are obtained by desorption of second layer molecules. The adsorption of BrP is self-limiting at room temperature. The coverages of diBr[4]H and bis[7]H are defined with respect to the integrated C 1s intensities of Br[7]H and Br[4]H which are expected to have a similar density of carbon atoms per unit area. Note different pass energies were used. Therefore the exact line shapes should not be compared.

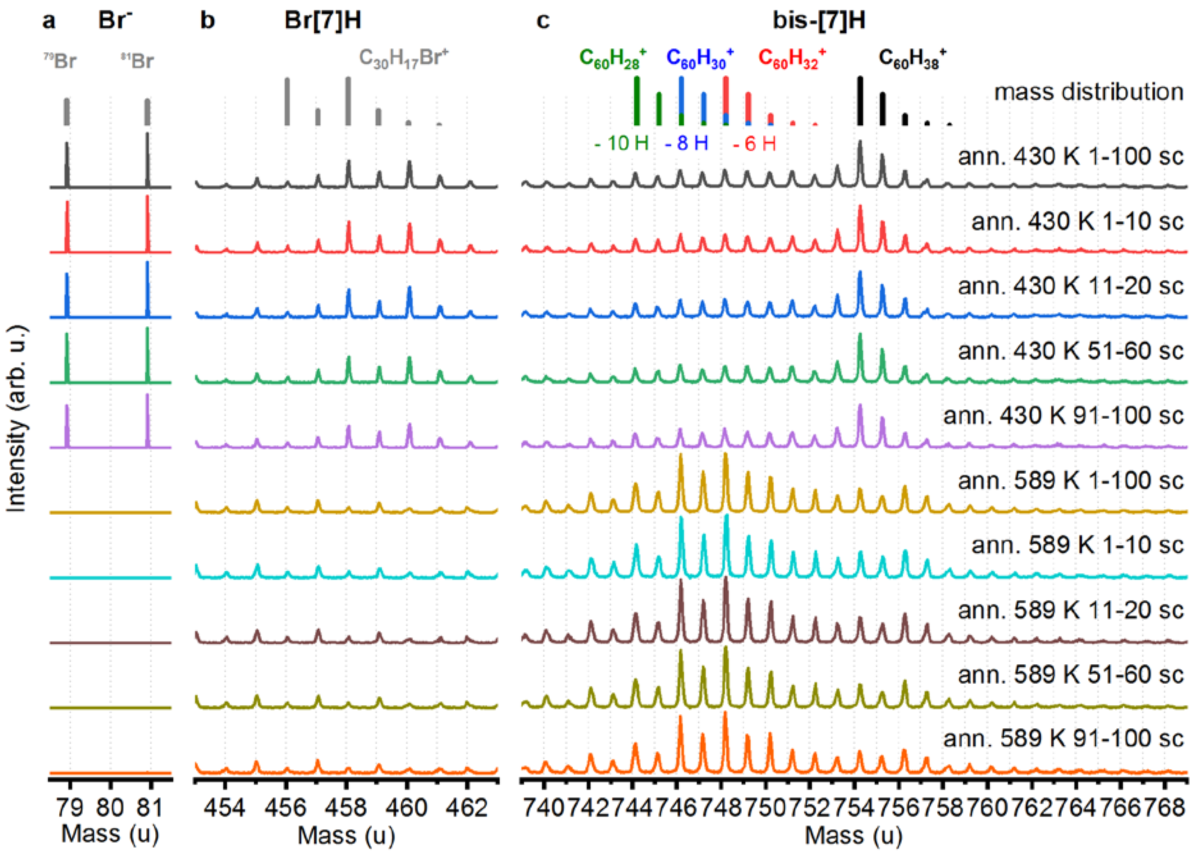

Figure S3: Evidence that the SIM spectra are in the static limit, i.e. that the dose of Bi_3^+ ions used during bombardment is low enough and does not affect the spectra. The ToF-SIMS data shown in Figure 3 and Figure S4 is used. Different subsets taken from the scan sequence (e.g. scans 1-10) are compared with each other and the total signal. The spectra are normalized to the number of scans. (a) Br^-, (b) Br[7]H ($C_{30}H_{17}Br^+$) and (c) bis[7]H mass regions.

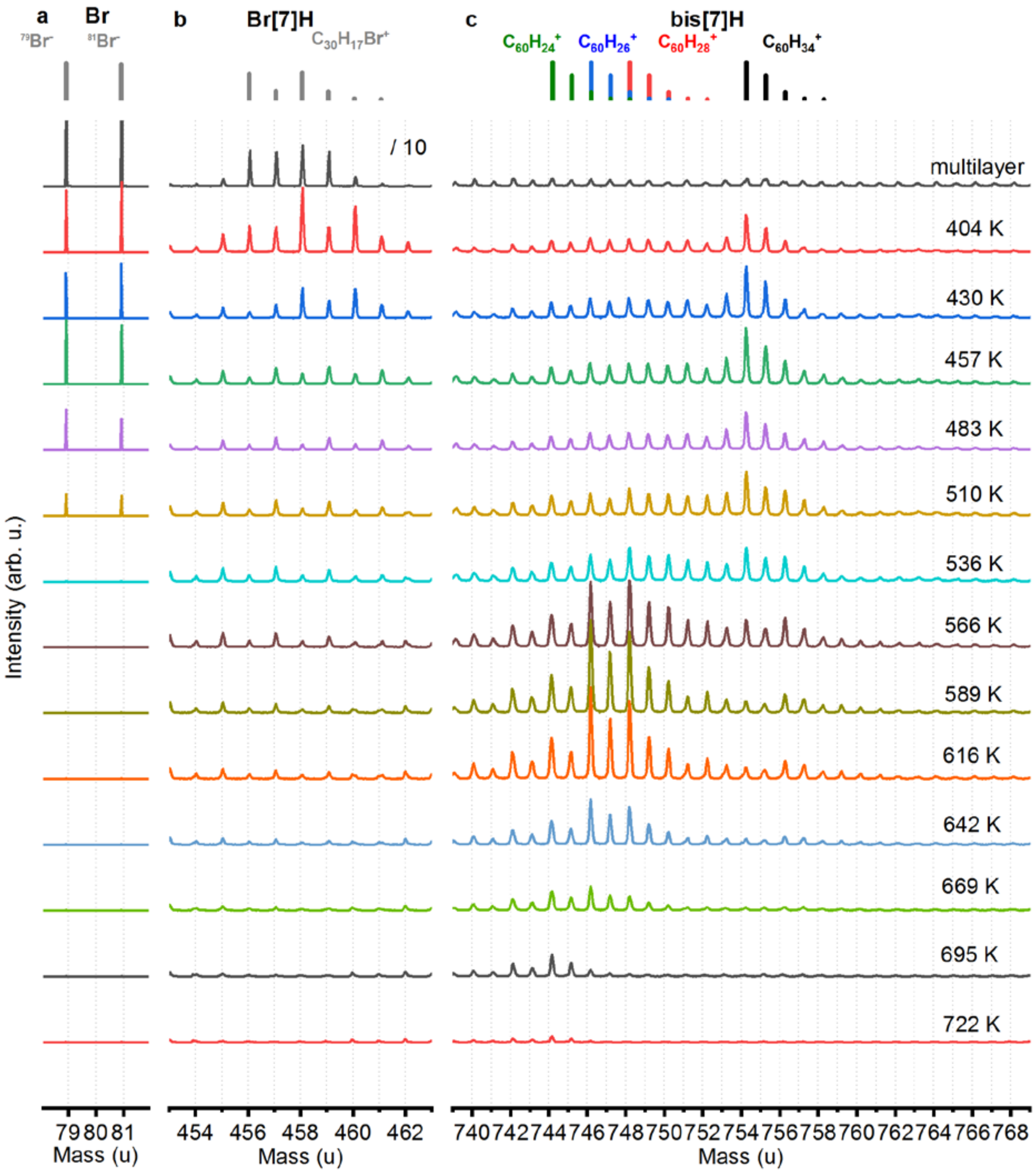

Figure S4: Complete set of the ToF-SIMS data shown in Figure 3, i.e. starting from a multilayer of Br[7]H on Au(111). (a) Br-, (b) Br[7]H (C30H17Br+) and (c) bis[7]H mass regions. The multilayer Br[7]H spectrum (only region in b) is divided by a factor of 10 for visibility.

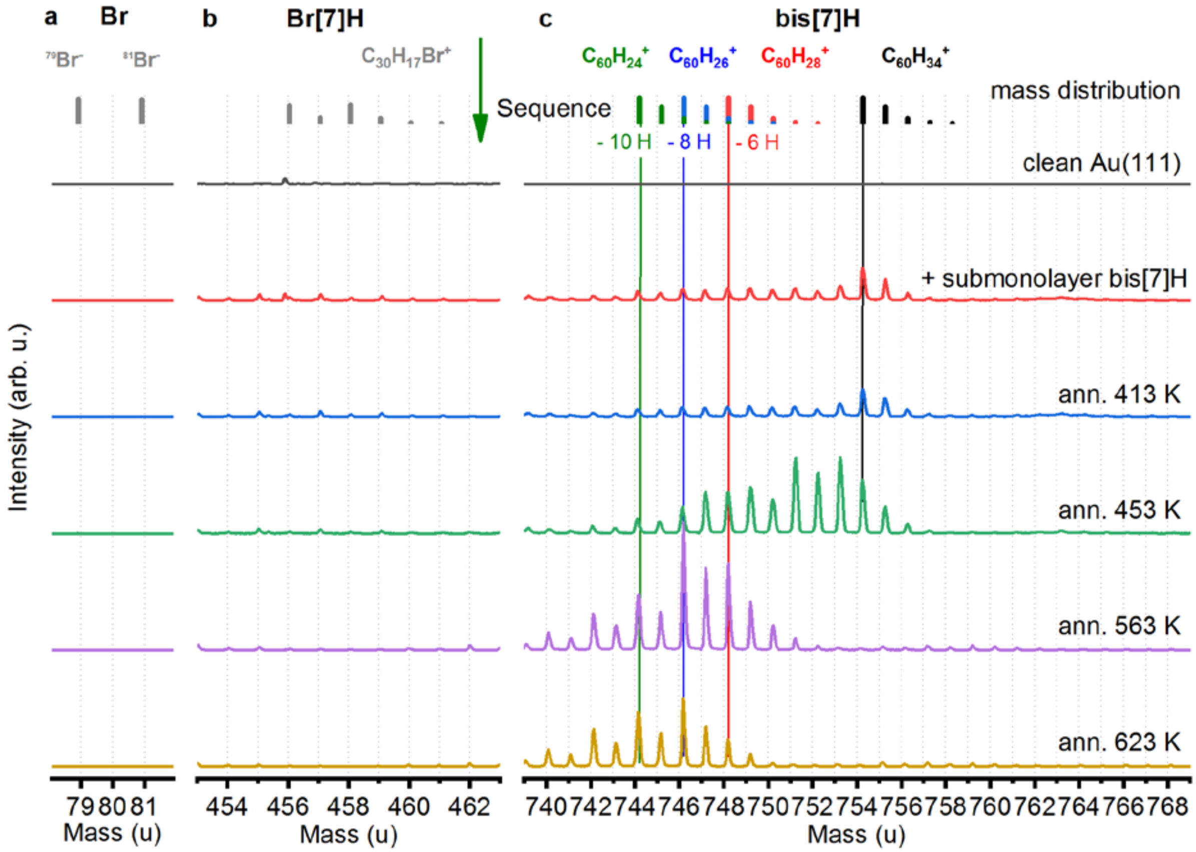

Figure S5: ToF-SIMS data obtained after annealing of approx. ¼ monolayer of solution-synthesized bis[7]H deposited on Au(111). (a) Br^-, (b) Br[7]H ($C_{30}H_{17}Br^+$) and (c) bis[7]H mass regions. No Br and Br[7]H is detected. The signal visible in Br[7]H region does not match the mass distribution of Br[7]H ($C_{30}H_{17}Br^+$), but corresponds to the fragmentation of bis[7]H during measurements. The dehydrogenation occurs in a similar way as in the case of Br[7]H and leads to formation of bis[7]H/–{6,8,10}H. This confirms that the presence of Br does not influence the dehydrogenation process of bis[7]H.

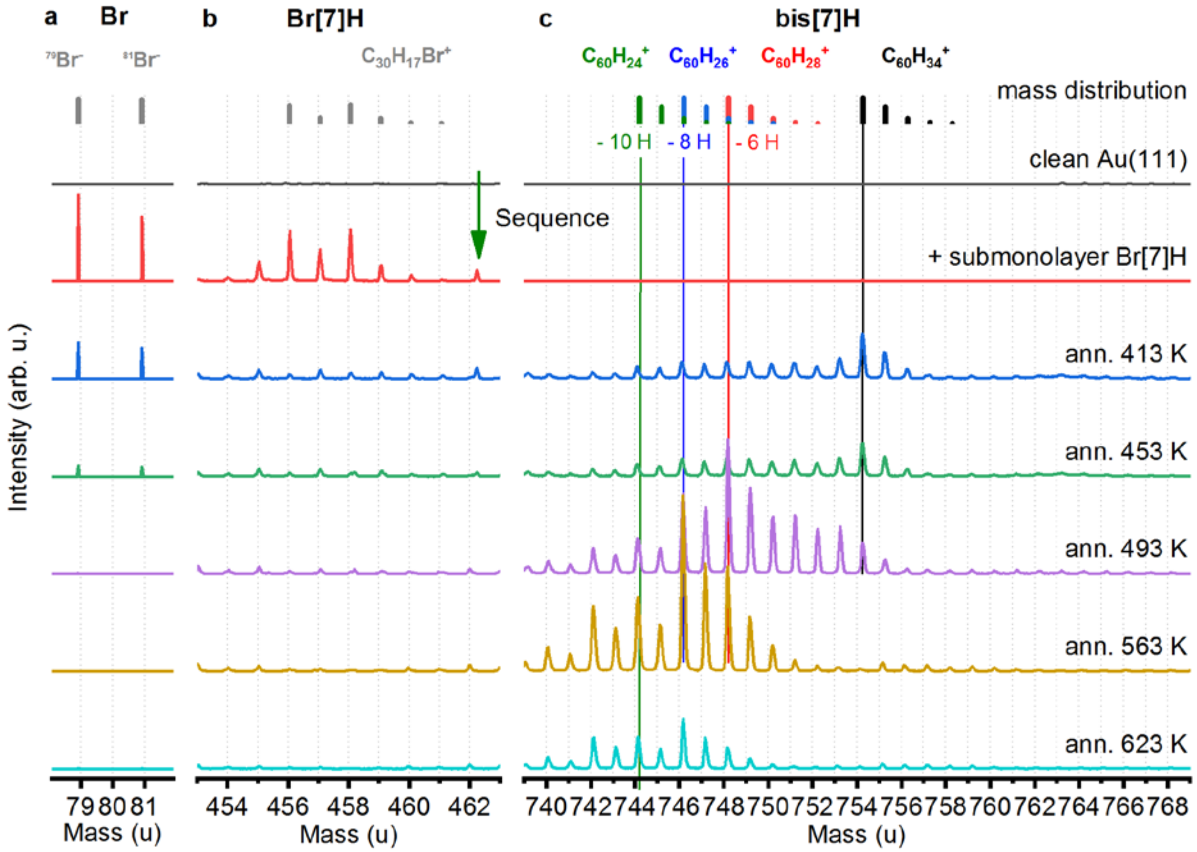

Figure S6: ToF-SIMS data of Ullmann coupling of approx. ¼ monolayer of Br[7]H on Au(111). (a) Br^{-}, (b) Br[7]H ($C_{30}H_{17}Br^{+}$) and (c) bis[7]H mass regions. Similar to the high coverage case, Br[7]H molecules are coupled to bis[7]H between 413 and 453 K, as confirmed by disappearance of the peaks representing Br[7]H and appearance of peaks representing bis[7]H. The Br signal is strongly reduced at 453 K and a very faint bis[7]H/–6H signal is detected. Major dehydrogenation is detected at 493 K.

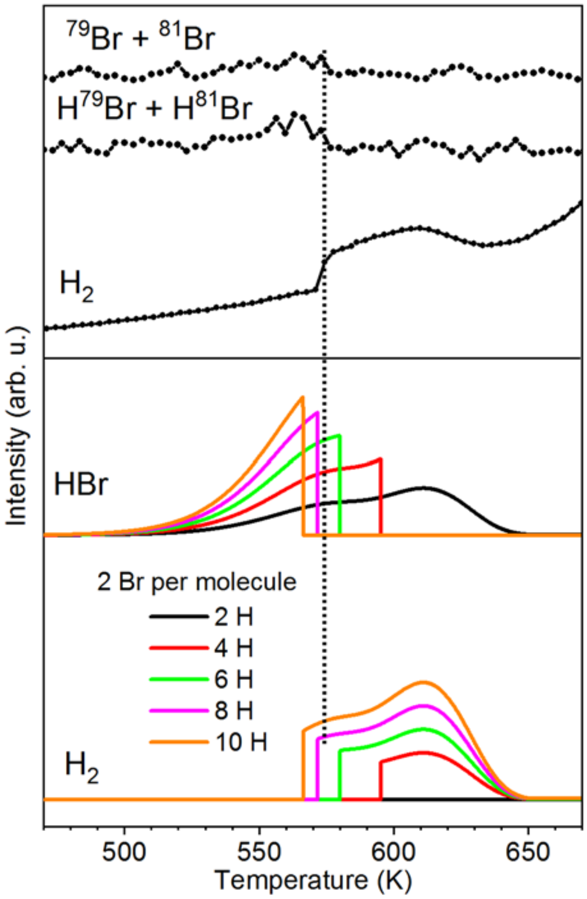

Figure S7: TPRS simulations considering different amounts of atomic H released per molecule. The number of H atoms is adjusted by scaling the coefficients in eq. S4 and by keeping the ratio H atoms released in the first and second dehydrogenation fixed to 1:2. The simulations are compared with the data shown in Figure 2c. The number of H atoms produced per molecule determines the position of the step. The simulations with 6, 8 and 10 H per molecule agree best with the data.

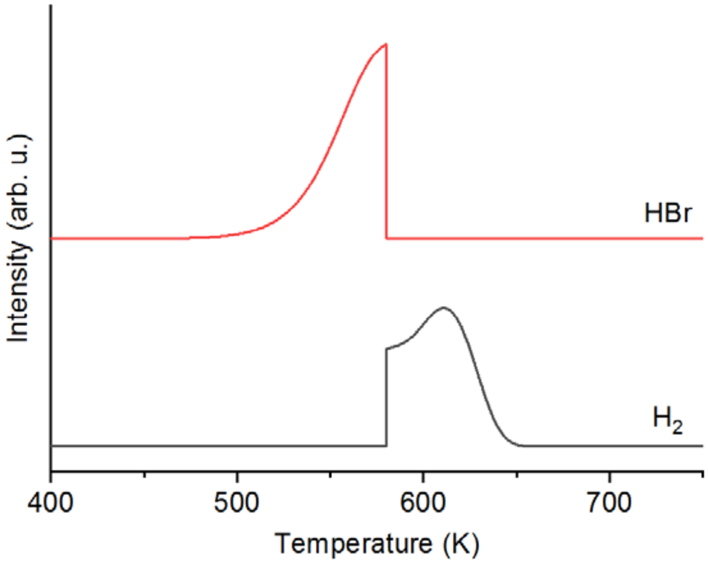

Figure S8: Simulations of HBr and H_2 evolution from 10,10'-dibromo-9,9'-bianthryl (DBBA). The dehydrogenation step is modelled as a single process $\frac{d\theta_{mol}}{dt}(\mathrm{t}) = -\nu\, \theta_{mol}\, e^{-\frac{E_{deH}}{RT}}$, with $E_{deH} = 162\frac{kJ}{mol}$ and $\nu = 10^{13}\ Hz$. Based on the stoichiometry of the molecule, the simulations were performed for 2 Br atoms and 8 H atoms released per DBBA molecule. The simulations are in quite good agreement with the TPR spectra published in ref. 1.

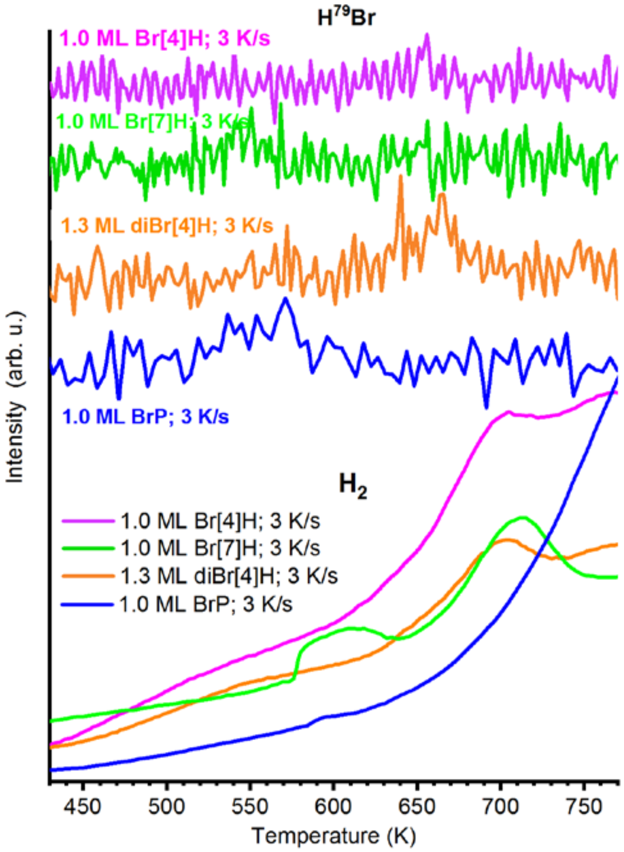

Figure S9: HBr (top) and H_2 (bottom) TPR spectra of saturated layers of different molecules. A clear H_2 TPR signal close to the HBr signal is only observed for Br[7]H. For bromopyrene a tiny H_2 signal is observed just after the HBr signal. Because dehydrogenation of bis-pyrene releases only 2 H atoms, all hydrogen can desorb as HBr. The HBr signal of Br[4]H and diBr[4]H is close to the unspecific dehydrogenation signal at ≈700 K. Non-specific dehydrogenation occurs at around 700 K for all molecules except Br-pyrene where it occurs at around 800 K.

References

1 C. Bronner, J. Björk and P. Tegeder, *J. Phys. Chem. C*, 2015, **119**, 486–493.
2 J. Li, K. Martin, N. Avarvari, C. Wäckerlin and K.-H. Ernst, *Chem. Commun.*, 2018, **54**, 7948–7951.
